# Phylogeography and population genetics of *Schizothorax o’connori*: strong subdivision in the Yarlung Tsangpo River inferred from mtDNA and microsatellite markers

**DOI:** 10.1038/srep29821

**Published:** 2016-07-18

**Authors:** Xiang-Zhao Guo, Gui-Rong Zhang, Kai-Jian Wei, Ruo-Jin Yan, Wei Ji, Rui-Bin Yang, Qi-Wei Wei, Jonathan P. A. Gardner

**Affiliations:** 1 Key Laboratory of Freshwater Animal Breeding, Ministry of Agriculture, College of Fisheries, Huazhong Agricultural University, Wuhan 430070, P. R. China; 2 Freshwater Aquaculture Collaborative Innovation Center of Hubei Province, Wuhan 430070, P. R. China; 3 Key Laboratory of Freshwater Biodiversity Conservation, Ministry of Agriculture, Yangtze River Fisheries Research Institute, Chinese Academy of Fishery Sciences, Wuhan 430223, P. R. China; 4 School of Biological Sciences, Victoria University of Wellington, P O Box 600, Wellington 6140, New Zealand

## Abstract

The Qinghai-Tibet Plateau (QTP) is a biodiversity hotspot, resulting from its geological history, contemporary environment and isolation. Uplift of the QTP and Quaternary climatic oscillations are hypothesised to have influenced the genetic diversity, population structure and dynamics of all QTP endemic species. In this study, we tested this hypothesis by assaying variation at two mitochondrial DNA regions (cytochrome *b* and control region) and at 12 microsatellite loci of seven populations of the endemic fish, *Schizothorax o’connori* from the Yarlung Tsangpo River (YLTR) on the QTP. Analyses revealed one group of six populations to the west, above the Yarlung Tsangpo Grand Canyon (YTGC), and a second group to the east below the YTGC. Estimates of the timing of this east-west split indicate that these groups represent evolutionarily significant units that have evolved separately and rapidly in the middle Pleistocene, at the time of the Kunlun-Huanghe Movement A Phase and the Naynayxungla glaciation. Population dynamic analyses indicate that *S. o’connori* experienced a pronounced late Pleistocene expansion during the last interglacial period. The results of this study support the hypotheses that the QTP uplift and Quaternary climatic oscillations have played important roles in shaping the population genetics and dynamics of this endemic fish.

Genetic diversity, the fundamental source of biodiversity, is a product of long-term evolution, with its distribution and extent in natural populations being mainly affected by gene flow, mutation, genetic drift and selection. The rapid development of molecular biology and the advent of new analytical approaches have dramatically improved our understanding of the evolutionary histories of many species, such that the contributions of landscape and ecological variation can now be linked to the extent and distribution of genetic diversity^1^,^2^.

Building on earlier work^3^, the last decade has seen substantial advances in our understanding of the importance of geological events such as regional uplift and isolation, ice ages and regressions, and the role of refugia in contributing to speciation events across many groups^4^,^5^,^6^. The contemporary distributions of species and their patterns of genetic structure at molecular markers that are maternally inherited, lack recombination, and exhibit rapid evolution (i.e. mitochondrial DNA, mtDNA), in combination with markers that are highly polymorphic and informative about contemporary gene flow (e.g. microsatellites) also provide new insights into anthropogenic processes that may erode patterns of genetic structure and barriers to gene flow within and between species^4^,^5^,^7^,^8^.

The Qinghai-Tibet Plateau (QTP) is the largest high-elevation ecosystem in the world. This region, along with southeast China and the Himalayan region, has been designated as one of the world’s 34 most important centres of biodiversity because of its high species richness and abundance of endemic species^9^. The QTP uplifted approximately 3000 m and experienced at least four major glaciations during the Pleistocene^10^. This uplift and associated climate changes are widely regarded as the most important factors influencing current spatial distribution, genetic diversity and population structure of local species^3^,^11^,^12^.

The Yarlung Tsangpo River (YLTR) is the largest river system on the QTP, with a catchment area of 238,000 km^2^ and a length of 2,091 km^13^. The YLTR originates from the Jimayangzhong Glacier (30°15′N, 82°21′E) on the northern slope of the Himalayas. In the vicinity of the eastern Himalayan syntaxis (about 95°E), the east-west flowing YLTR makes an abrupt, tight loop. The river cuts through the Himalayas in the Yarlung Tsangpo Grand Canyon (YTGC) which is the deepest gorge on earth^13^. Within a region known for high biodiversity, the YLTR basin is notable for high species richness and the abundance of endemic fish species.

Fishes of the genus *Schizothorax* (Teleostei: Cyprinidae), also collectively known as snow trout, number more than 60 valid species from central and eastern Asia. In China, more than 30 *Schizothorax* species are widely distributed in different rivers and lakes on the QTP and its adjacent area^14^,^15^. *Schizothorax o’connori* Lloyd 1908 is an endemic tetraploid fish that is distributed in the main streams and tributaries along the upper and middle reaches of the YLTR in Tibet^16^. There are seven endemic fishes in the YLTR, of which five are limited to the western drainages above the YTGC and only two, *Schizothorax o’connori* and *Schizopygopsis younghusbandi*, are thought to be distributed in both the western and eastern drainages of the YLTR^13^,^17^. As such, *S. o’connori* is an excellent model organism to examine the impact of uplift of the QTP and Quaternary climatic oscillations on the population structure and dynamics of species endemic to the QTP.

Genetic diversity and population genetic structure across a species’ range reflect historical events such as range contraction and expansion (recolonisation) from glacial refugia, as well as current habitat fragmentation and dispersal^8^. During the middle and late Pleistocene, glacial and interglacial periods caused repeated changes to the distributions of endemic fishes on the QTP. In the glacial periods, schizothoracine species persisted primarily in refugia in lower altitude valleys and tributaries. Interglacial periods enabled these species to expand to higher valleys and tributaries, with leading-edge populations carrying a subset of the genetic diversity of refugial populations. This rapid range expansion is associated with periods of speciation, leading to high levels of endemism. Simultaneously, range expansion to higher valleys and tributaries in the west resulted in populations there having reduced connectivity with the eastern populations. This, in turn, resulted in increased regional diversification, leading to increased genetic uniqueness (e.g. private alleles) or even endemism^13^. In addition, huge gorges (such as the YTGC) and waterfalls were formed with the rapid and intensive uplift of the QTP. Such features became a barrier to exchange between the western and eastern drainages and prevented gene flow between the two regions^13^. To date, all of the biogeographic and phylogenetic studies of the schizothoracine fishes on the QTP are based on the results of only one mtDNA gene/sequence^13^,^15^,^18^ or two mtDNA gene/sequences^4^,^5^,^19^. No study has employed both mtDNA gene/sequences and nuclear DNA microsatellites to investigate the population genetics and demographic histories of the QTP endemic schizothoracine species.

In this study, levels of mitochondrial and nuclear DNA genetic variability and population genetic structure of *S. o’connori* from seven populations were investigated using mitochondrial cytochrome *b* (Cyt *b*) and control region (CR) sequences and twelve polymorphic nuclear microsatellite loci. The primary aim of our study was to test the hypothesis that the population structure and dynamics of *S. o’connori* are influenced by (coincident in timing with) the uplift of the QTP and Quaternary climatic oscillations. Secondarily, our aim was to compare the genetic uniqueness of *S. o’connori* in the western region with that in the eastern region to better understand the interplay between geological upheaval, geographic isolation and genetic diversity. Our results shed new light on the evolutionary history of the endemic QTP fish fauna and will also aid in the conservation of endemic fishes of this region.

## Results

### Mitochondrial DNA (mtDNA) sequence data, genetic diversity and population structure

Full Cyt *b* and partial CR (control region) sequences were successfully amplified from 168 samples of *S. o’connori*. Cyt *b* (1141 bp) had 84 variable sites, 59 of which were parsimony informative: all variable sites were transitions, except one transversion. CR sequences were ~714 bp long and contained 67 variable sites, 47 of which were parsimony informative, with one indel: all variable sites were transitions, except two transversions. Because the Cyt *b* and CR regions have not reached saturation they are suitable for analyses employed here. The partition homogeneity test indicated no incongruence between the Cyt *b* and CR sequences; we therefore focussed on a concatenated mtDNA sequence of 1855 bp (5′-Cyt *b*-CR-3′) for analysis. The Cyt *b* and CR haplotypes were defined and their sequences submitted to GenBank (Accession numbers KT188614 – KT188672 for Cyt *b* and KT188673 – KT188752 for CR).

**Figure 1:**
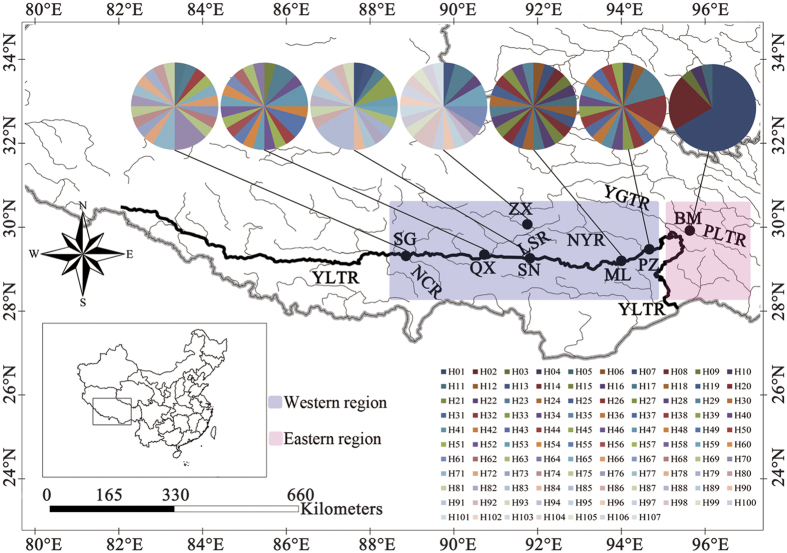
Sampling locations, geographical distribution of 107 Cyt *b* + CR haplotypes and two clades identified in *Schizothorax o’connori* on the Qinghai-Tibet Plateau (western region – YLTR; eastern region – PLTR). YLTR, Yarlung Tsangpo River; PLTR, Parlung Tsangpo River; YGTR, Yigong Tsangpo River; NCR, Nianchu River; LSR, Lhasa River; NYR, Nyang River. SG – Shigatse; ZX – Zhaxue; QX – Quxu; SN – Shannan; ML – Mainling; PZ – Paizhen; BM – Bomi. The map was drawn using ArcGIS 10.2 (ESRI, CA, USA) and Adobe Photoshop CS5.1 (Adobe Systems Inc., CA, USA).

**Figure 2:**
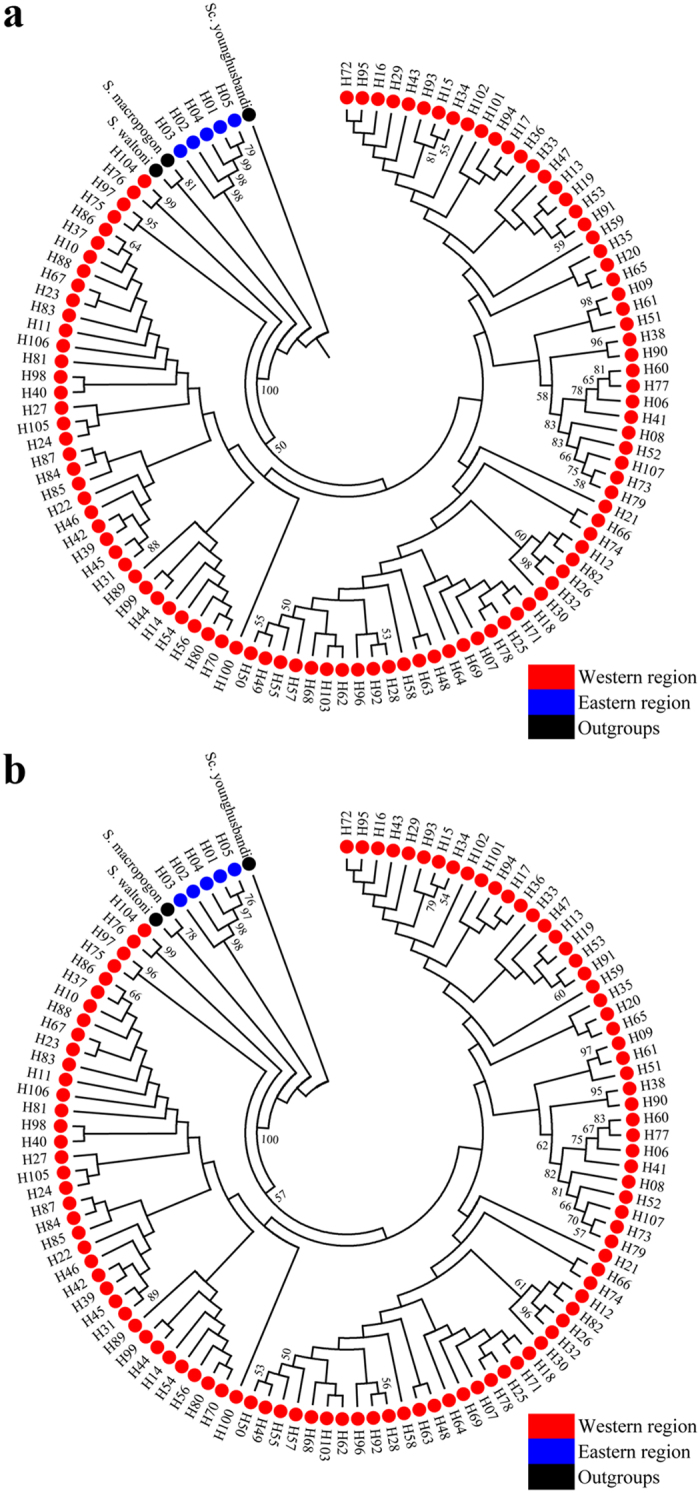
NJ (**a**) and ML (**b**) phylogenetic trees of *Schizothorax o’connori*, based on concatenated mtDNA sequences (Cyt *b* + CR) haplotypes. The numbers above the branches correspond to bootstrap support >50% obtained in the NJ and ML analyses.

In total, 107 mtDNA haplotypes (Fig. 1; Supplementary Table S1) were observed in the concatenated Cyt *b* + CR sequences. The neighbour-joining (NJ) and maximum likelihood (ML) phylogenetic methods resulted in trees with similar topologies (Fig. 2). Two main groups were recognised, a western group containing haplotypes from Shigatse, Zhaxue, Quxu, Shannan, Mainling and Paizhen, and an eastern group containing five private haplotypes from the Bomi population. In the western group, no monophyletic groups were observed. Only two haplotypes (H76 and H104) from Shigatse or Zhaxue formed a sister group with other haplotypes from the western group. Similar results (western and eastern groups) were found based on NJ and ML phylogenetic analyses of Cyt *b* and CR haplotypes separately (Supplementary Figs S1 & S2). All the Cyt *b* and CR haplotypes from the six western populations formed a group and all four Cyt *b* haplotypes (H56–H59) plus the four CR haplotypes (H77–H80) from the eastern population (Bomi) formed another group. Interestingly, haplotypes from fish of the western region grouped firstly with outgroups (*S. waltoni* and *S. macropogon*) and then grouped with the haplotypes from the eastern region (Fig. 2).

**Figure 3:**
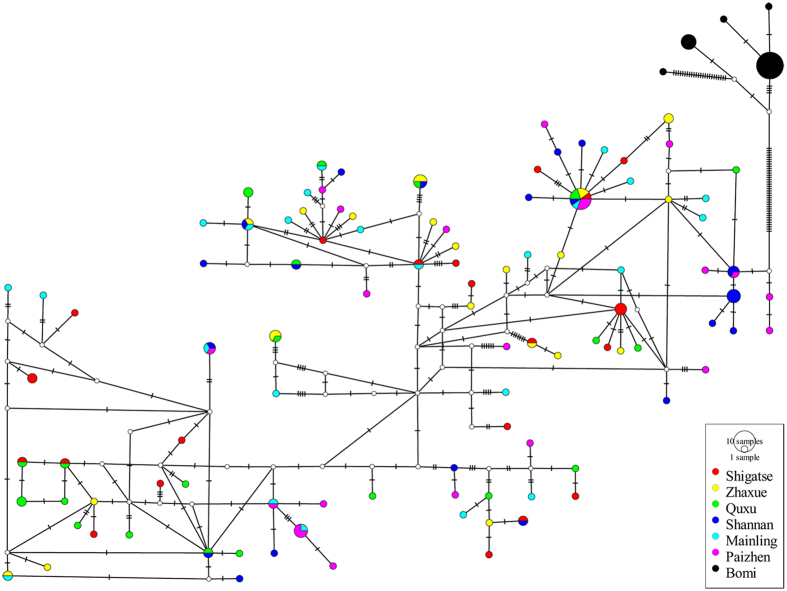
Median-joining network of concatenated mtDNA sequences (Cyt *b* + CR) haplotypes from seven populations of *Schizothorax o’connori*. The circle size of haplotype denotes the number of observed individuals. Colors correspond to different regions. White circles represent intermediate haplotypes not observed.

The median-joining networks (Fig. 3) identified two main mtDNA haplogroups corresponding to the western and eastern regions, with no mtDNA haplotypes shared between them. There were 17 haplotypes shared by two or more western populations, and each western population had one or more private haplotypes (Supplementary Table S1). The most abundant haplotype, Hap23, was present in all six western populations (Fig. 3, Supplementary Table S1), suggesting that it is ancestral. Five haplotypes (Hap01 to Hap05) were observed only in the Bomi population from the eastern drainage (Fig. 3, Supplementary Table S1). The western populations were characterized by high haplotype and low nucleotide diversity values, whereas the eastern population had low haplotypic and low nucleotide diversity values (Supplementary Table S2). Similar median-joining networks and population genetic diversity results were identified from the Cyt *b* and CR analyses (Supplementary Figs S3 & S4, Table S2).

The Kimura 2-parameter (K2P) distances and the pairwise *Φ*_ST_ values in the west were small; much larger distances were observed between the east and west regions (Supplementary Table S3). Analysis of molecular variance (AMOVA) results based on concatenated mtDNA sequences indicated that significant variance occurred among and within populations. When analysed by region (west/east) and populations, AMOVA revealed non-significant variance between regions (west/east), whereas significant variance occurred within populations and among populations within regions (Table 1).

Tests of isolation by distance (IBD) based on concatenated mtDNA sequence were not statistically significant for all seven populations (*R* = 0.222, *P* = 0.255), for the six western populations (*R* = 0.172, *P* = 0.227). IBD tests were no significant for all seven populations or the six western populations based on Cyt *b* only and CR only (results not shown here).

### Divergence time of cladogenesis and demographic history based on mtDNA sequences

**Figure 4:**
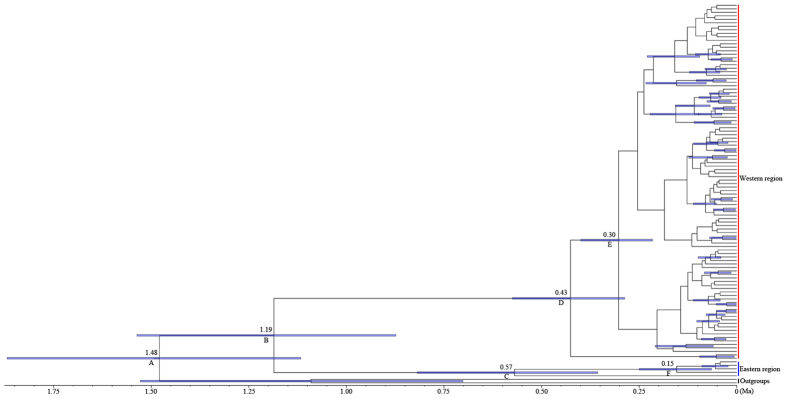
Bayesian phylogenetic tree for *Schizothorax o’connori*, based on concatenated mtDNA sequences (Cyt *b* + CR) haplotypes. The numbers above the branches are the estimates of divergence times (million years, Ma) within *Schizothorax o’connori* for the major nodes by BEAST analysis. Blue shaded bars indicate the 95% highest posterior density (HPD) for node ages and scale bar represents time in millions of years from the present day. Western and eastern clades are denoted by red and blue lines, respectively.

**Figure 5:**
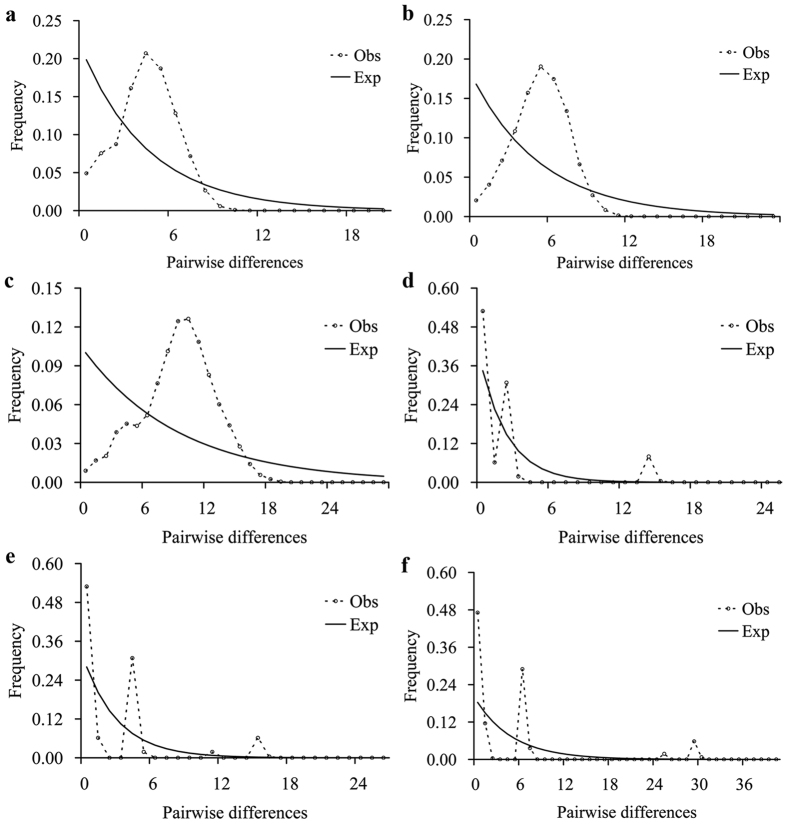
Observed and expected mismatch distributions for *Schizothorax o’connori* in the western region for (**a**) Cyt *b*, (**b**) CR and (**c**) Cyt *b* + CR, and in the eastern region for (**d**) Cyt *b*, (**e**) CR and (**f**) Cyt *b* + CR. Dashed line, observed distribution; solid line, theoretical expected distribution under a population expansion model.

**Figure 6:**
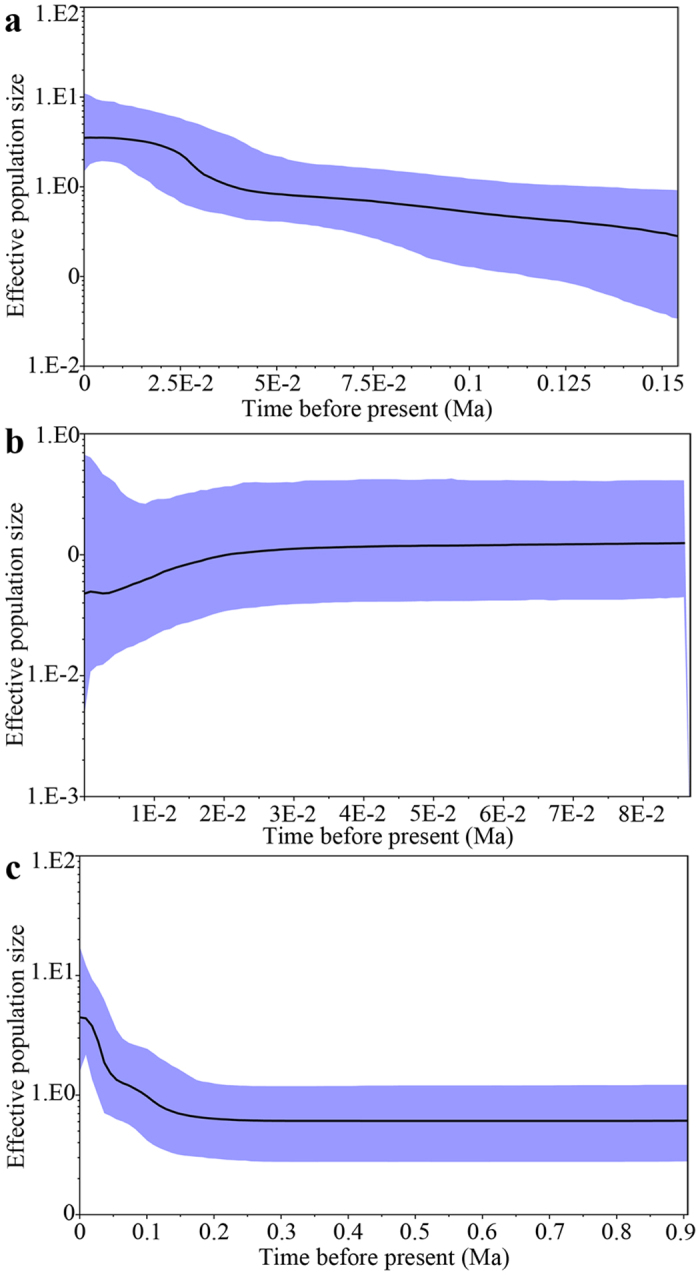
Bayesian skyline plots (BSPs) estimated by BEAST for *Schizothorax o’connori* in the west region (**a**), east region (**b**) and both west and east region (**c**). The *X*-axis shows time in millions of years before present. The *Y*-axis (logarithmic scale) indicates effective population size of females (N_e_) estimates multiplied by generation time (T_gen_). The solid line indicates the median of population size, and the 95% HPD credibility interval is depicted in blue.

Divergence time between the western and eastern regions based on the combined data set (Fig. 4, node B, 1.19 Ma) was close to that determined for Cyt *b* alone (Supplementary Fig S5, node B, 1.18 Ma), and places the timing of the event during the mid-Pleistocene. Unimodal mismatch distributions (Fig. 5a–c) and significant negative values for Fu’s *F*s (Supplementary Table S2) were observed for the six western populations. The sum of squared deviations (*SSD*) and raggedness index values (Supplementary Table S2) supported a sudden expansion model for the western populations. For the Bomi population (eastern region), multimodal mismatch distribution patterns (Fig. 5d–f) and non-significant positive values for Fu’s *F*s index (Supplementary Table S2) were observed: the expansion hypothesis was rejected. Bayesian skyline plot (BSP) analyses supported the hypothesis of population expansion in the western region (Fig. 6a) and population stability in the eastern region (Fig. 6b) from the late Pleistocene onwards. For the western region, a marked demographic expansion began shortly after the most recent common ancestor shared by the east and west regions and continued until the recent past. In contrast, the BSP for the eastern region showed no marked demographic changes for a long time and then a recent change. Based on all populations in the western and eastern regions, BSP analysis showed that the populations had no marked demographic changes for a long time and then ~0.10 Ma they experienced a pronounced population expansion (Fig. 6c). Expansion time was estimated to be approximately 0.098 Ma (0.087–0.114 Ma, Table 2) based on the equation t = τ/2u.

### Genetic diversity and population structure based on microsatellite data

All 12 microsatellite loci displayed up to 4 bands per individual, as expected for a tetraploid genome, and were highly polymorphic (Supplementary Table S4). Population genetic diversity statistics in the western region were generally larger than those in the eastern region (Table 3). Of the 359 bands observed, 288 bands (69%) were shared by all populations: the remaining bands were population-specific (private). All the western populations above the YTGC had similarly high values of percentage of polymorphic loci (*PPL*), Nei’s gene diversity (*H*) and Shannon’s information index (*I*). The eastern population of Bomi had low observed values compared to the western populations.

All population pairwise *F*_ST_ values were significant after Bonferroni correction for multiple testing, except that between Shigatse and Paizhen. *F*_ST_ values and Nei’s unbiased genetic distance values between pairs of western populations were generally lower than those between Bomi and any population in the western region (Table 4). AMOVA results indicated that 8.37% of the variance was maintained among populations and 91.63% within populations (Table 1). When analysed by region (west/east) and populations, 9.30% of the variance was between regions, whereas 85.54% occurred within populations and 5.16% occurred among populations within regions (Table 1).

**Figure 7:**
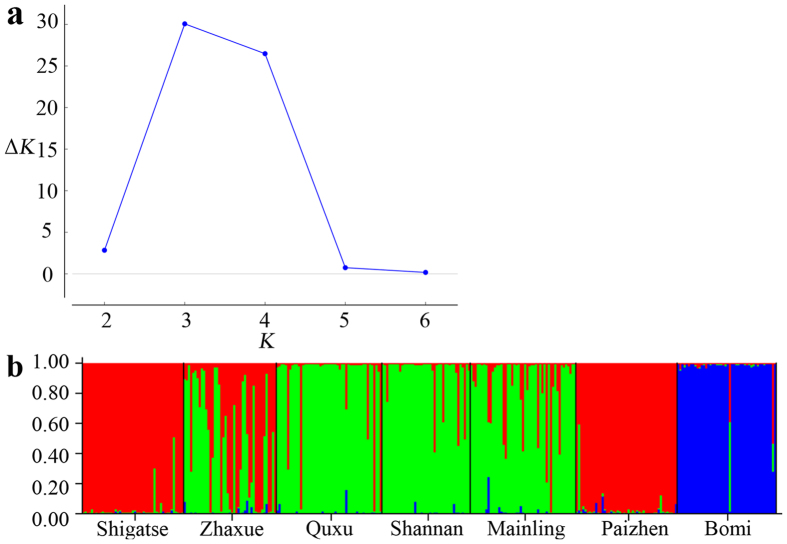
The model choice criterion ln*P*(D) of the STRUCTURE analysis for each *K* value (**a**) and STRUCTURE analysis of microsatellite locus variation in all *Schizothorax o’connori* populations (**b**).

**Figure 8:**
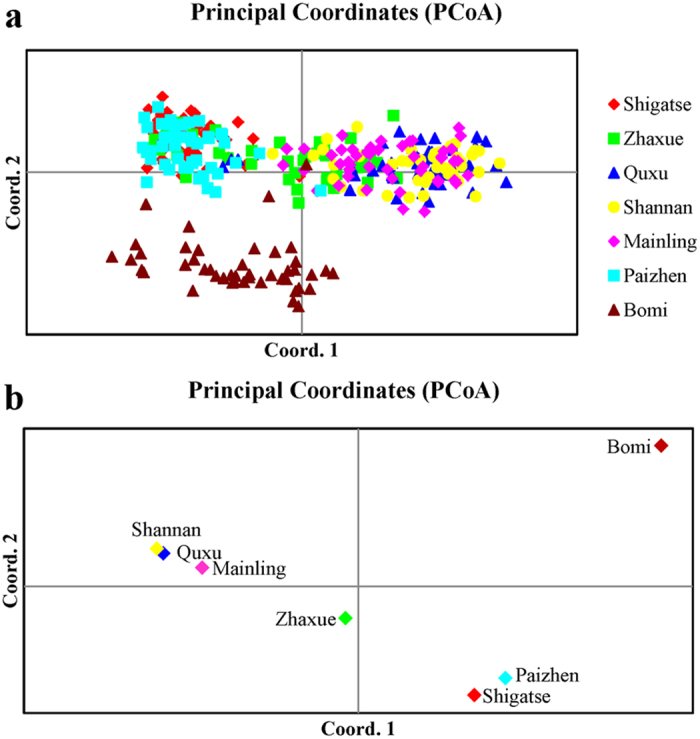
Principal coordinate analysis (PCoA) of pairwise distances between individuals (**a**) and populations (**b**) of *Schizothorax o’connori*.

STRUCTURE and the Δ*K* method inferred *K* = 3 groups (Fig. 7a,b). The six populations in the western region were divided into two subgroups: almost all individuals of Shigatse, Paizhen and some samples of Zhaxue were assigned to one subgroup (Red), whilst the majority of individuals of Quxu, Shannan, Mainling and some samples of Zhaxue were assigned to another subgroup (Green). In the east, almost all individuals of Bomi were assigned to a third (eastern) group (Blue). Similar results were also found in the PCoA analyses (Fig. 8a,b).

Tests of isolation by distance (IBD) were not statistically significant for all seven populations (*R* = 0.253, *P* = 0.180) or for the six western populations (*R* = 0.061, *P* = 0.340).

## Discussion

This study brings together, for the first time, analyses of mitochondrial and nuclear DNA variation to better understand how processes such as the uplift of the QTP and Quaternary climatic oscillations may influence demographic history and population genetic structure among a speciose group of fishes on the QTP. Consistent with other such studies we have based our estimates of the timing of the split between the eastern and western regions, as well as the timing of the population expansion within the western region, on the use of mitochondrial markers^4^,^5^,^15^,^18^,^19^. Whilst nuclear DNA sequence data (e.g., ITS) may have bolstered the mitochondrial DNA-based findings, we note that the results from the nuclear DNA microsatellite markers have confirmed the east-west split, but cannot help with the timing of the split or the timing of the population expansion. The molecular clock (substitution rate) estimates associated with the mtDNA Cyt *b* and the CR sequences provide slightly different timings for the split, but this is to be expected given the coding and non-coding functions of these two markers, respectively. Nonetheless, the difference in estimates is not great and supports the use of the concatenated DNA sequence. As noted above, our estimates of the timing of the split and for the population expansion out of the presumed western refugia for *S. o’connori* are in strong agreement with other such estimates derived for a range of different fishes, and they are supported by the timing of major geological events reported for the QTP.

### Evolutionary history of western and eastern populations

The demographic history and divergence time of *S. o’connori* populations are explained by the palaeogeographical history and palaeoclimatic oscillations of the QTP, as well as by the geological structure and isolation of the contemporary environment. Based on mtDNA analysis there is strong evidence in the six western populations on the YLTR of spatial or demographic expansions^4^,^18^,^20^. The scenario in the west (on the YLTR) contrasts with that in the east (on the Parlung Tsangpo River, PLTR) where mtDNA analysis indicates a stable situation that is characterised by the absence of a recent bottleneck and no evidence of population expansion. Results from the BSP and the mismatch distribution equation (t = τ/2u) indicate a time of population expansion of *S. o’connori* in the west of ~0.1 Ma (0.087–0.114 Ma, Late Pleistocene) which was similar to the previous results ~0.125 Ma (0.093–0.145 Ma)^13^. The reason for the slight difference between these two estimates is likely to be due to the inclusion of CR sequence data in addition to the Cyt *b* data, for our work, and the use of only Cyt *b* in the earlier work^13^. Interpretation of the situation in the east has to be understood in the context of having only one population (Bomi). Thus, our study is unlikely to have captured all of the genetic diversity present in this region, with consequent effects on our estimates of the time of the east-west (YLTR-PLTR) divergence and also of the specific status of the fishes from Bomi (discussed in subsequent section). Our original study was designed to assess the genetic structure and connectivity of *S. o’connori* populations on the QTP. The subsequent identification of the pronounced east-west divergence, even if based on only one eastern population, is novel and requires interpretation and explanation.

The divergence time of the *S. o’connori* populations between the western (the YLTR) and eastern (PLTR) regions was estimated to be 1.19 Ma. This divergence date, which as noted above is based on only one eastern population, is associated with the Kunlun-Huanghe Movement A Phase (about 1.1 Ma) in the mid-Pleistocene^21^. Before the middle Pleistocene the critical elevation of the QTP was about 2000 m, and the QTP reached its present height (~3000 m) more recently, during the later Kunlun-Huanghe Movement (about 0.6–1.1 Ma) and the Gonghe Movement (~0.15 Ma)^22^. The formation of the YTGC and the four other great waterfall systems that range from 15 to 35 m in height in the region, probably occurred during the Kunlun-Huanghe Movement^12^,^13^,^23^, that is, at approximately the same time as the east-west split of *S. o’connori*. The YTGC formed a barrier between the western and eastern regions and prevented the dispersal of fish and resulted in isolation between populations on the two sides. The YTGC clearly forms a barrier to fish trying to migrate up the river (in a westerly direction), and whilst, in principle, fish may move down the river (in an easterly direction) although there is little or no evidence to indicate that this happens (see subsequent discussion of possible hybridisation). The Kunlun-Huanghe Movement has shaped the geomorphology of the area, making the topography of the YLTR very complex, with periodic uplift and repeated glacial-interglacial changes having a significant impact on the environment and climate of the QTP^12^. It therefore seems highly likely that the initial divergence of *S. o’connori* was shaped by the uplift events of the QTP and the associated climate change. The expansion and evolution of *S. o’connori* are therefore associated with the environmental changes and the violent upheaval of the QTP^14^, and the date of the separation between the east and west regions aligns with the Kunlun-Huanghe Movement A Phase (about 1.1 Ma)^21^. This interpretation is important in the context of understanding the evolution and maintenance of the high levels of mitochondrial and nuclear DNA diversity identified in *S. o’connori*, in particular in the west.

The distribution of *S. o’connori* in the east extends from the YTGC to Bomi (elevation ~2685 m) and covers the PLTR basin, which is the biggest tributary of the YLTR. However, some studies indicated that the PLTR was not the eastern tributary of the YLTR prior to 4 Ma, but the main stream of the YLTR in the east^24^. Based on the age of uplift of the Namche Barwa massif 25, it has been suggested that the palaeo-YLTR was captured by either the Lhuit River or the Brahmaputra River^24^. Similar results were reported in the biogeography and molecular phylogeny of the genus *Schizothorax*^15^ and phylogeography of *Buddleja crispa*^26^. These results indicate that *S. o’connori* was formerly distributed in the PLTR (east) and in the YLTR (west). With river capture, reversal events and uplift of the QTP^15^,^24^,^26^, *S. o’connori* populations in the east and west were gradually isolated from each other.

The estimate of the timing of population expansion (~0.1 Ma) reported in this study is consistent with the timing of the last interglacial that occurred 0.070–0.130 Ma^27^. Because of extensive ice coverage during the period of the penultimate glaciations, *S. o’connori* (and other QTP river fishes) only lived in restricted areas in lower valleys of the western region, but once the glaciers retreated (during the last interglacial period), *S. o’connori* expanded from restricted areas in the lower valleys of the YLTR to newly available habitat in the upper reaches of the river. The lower valleys and rivers in the western region of the YLTR are therefore likely to have been refugia for *S. o’connori* during the glacial period. The estimates of the expansion period in this study indicate that *S. o’connori* was present in the western region (e.g. Shigatse and Lhasa River) of the YLTR by the middle to late Pleistocene (~0.1 Ma). Thus, the demographic history of *S. o’connori* has been profoundly influenced by the Quaternary climatic oscillations. Consistent with the sequence of geological events in the region, the population expansion time estimate for *S. o’connori* is similar to that for other schizothoracine fishes living on the QTP (0.11 Ma for *Schizopygopsis pylzovi*^18^, and 0.057–0.108 Ma for *Gymnocypris* fishes^4^, but a little earlier than *Gymnocypris chilianensis*^5^ (0.033–0.055 Ma), and a little later than for *Schizopygopsis younghusbandi*^19^ (0.25–0.46 Ma). The difference of population expansion times among various schizothoracine fishes may be related to their different demographic histories, population sizes and dispersal abilities^12^.

### Population genetic variation

Analyses of mtDNA sequence and microsatellite variation in *S. o’connori* reveal pronounced population genetic structure at different hierarchical levels and a remarkable association with the two major geographic groups in the west and east. Whilst the mtDNA sequence and nDNA microsatellite results suggest that *S. o’connori* exhibits high levels of genetic variation in the west, in the east the Bomi population is characterised by low levels of genetic variation but a large number of private alleles. In the west, the Paizhen, Shigatse and part of the Zhaxue populations form one subgroup, whereas the Quxu, Shannan, Mainling and part of the Zhaxue populations form a second subgroup. There is no geographic underpinning for these two subgroups, with Paizhen being at the eastern end of the YLTR, Shigatse being at the western end of the YLTR, and with Zhaxue being on the Lhasa River, which is a tributary of the YLTR. The status of the Zhaxue population suggests that it is a mixture of the two subgroups within the YLTR. The Paizhen and Shigatse populations, which are geographically distant from each other, show a surprising degree of genetic similarity based on microsatellite variation. There is no obvious explanation for this, although we can rule out natural gene flow because this would be expected to affect populations in between these sites along the river. The most likely explanation for this anomalous result is human-mediated movement of fish from Shigatse to Paizhen, as part of a government-funded breeding programme set-up approximately 10 years ago (pers. obs. of the authors). Whilst it is impossible to confirm if human-mediated movement of fishes has occurred and that it has resulted in genetic mixing of different regional genotypes, we note that a similar pattern of population genetic similarity that is not related to geography (i.e., cannot be explained by river flow) has been reported for another fish (*Glyptosternum maculatum*) from the same river system^28^,^29^. Such a finding is consistent with human-mediated movement of fishes but does not prove it. The collection of further population samples from the eastern region to supplement the one sample from Bomi would be informative. However, fish numbers and mean fish size in the eastern region are in decline (pers. obs. of the authors) and the eastern region is particularly difficult to access. The recognition of two groups (west/east phylogroups) based on pronounced mtDNA and nDNA differences is consistent with previous findings for this fish^13^, but with the addition of the new analyses presented here may now require a reconsideration of the systematic status of the eastern group (see later).

The western populations of *S. o’connori*, spanning ~700 km from Shigatse (elevation ~3842 m) to Paizhen (elevation ~2922 m), constitute a single group characterised by high genetic diversity and gene flow. In the western region, mtDNA analyses revealed high *Hd* and low *π* values for each population. Such results can be attributed to rapid population expansion after a period of small effective population size^30^,^31^. Rapid population growth has enhanced the accumulation of mutations, and the expansion time was sufficient to permit generation of high haplotypic diversity, but insufficient for an increase in nucleotide diversity^18^. Many fishes with high *Hd* and low *π* values have originated in the Pliocene or early Pleistocene, but their mtDNA genealogies coalesce on a more recent time scale, perhaps the last few hundred thousand years^20^. Our results are consistent with reports for other fishes from the QTP^4^,^5^,^19^, all of which point to rapid and pronounced population expansion in the Pleistocene.

### Status of fishes in the eastern region

Following river capture, reversal events and uplift of the QTP^15^,^24^,^26^, the *S. o’connori* populations have been gradually isolated from each other, such that populations in the east (PLTR) and west (YLTR) have evolved independently and formed the genetic structure observed today. Whilst additional studies of anatomy, physiology and molecular biology are required to evaluate the systematic status of the eastern group, there is now clear evidence of profound nuclear and mitochondrial DNA differentiation consistent with inter-specific differences between the eastern and western groups. Whilst data from only one eastern population limits inferences that can be drawn, the low mtDNA sequence diversity and the low nDNA allelic diversity estimates^18^ both strongly suggest that the Bomi population has experienced periods of low effective population size within the recent past (last few tens of thousands of years).

All analyses of mtDNA and nDNA point to substantial differences between the eastern (Bomi) population and the six western populations of *S. o’connori*. Barcoding of Cyt *b* and COI are effective for species identification in freshwater fishes^32^. Although no explicit standards have been reported to identify species based on Cyt *b* sequences, a COI average intraspecific K2P distance of 0.35% and an average congeneric K2P distance of 8.11% have been employed as the standard to identify fish species^32^. Elsewhere, a COI average K2P distance of 1.75% between two *Schizothorax* species from the western Himalaya region (India) has been reported^33^. The high K2P distances reported here between the western and eastern regions indicate that the eastern population that is currently considered as *S. o’connori* might be classified as a subspecies or a new species in the genus *Schizothorax*. This interpretation is consistent with similar high mtDNA Cyt *b* + CR K2P distances reported between subspecies of *Cyprinus carpio*^34^. A review of the specific status of the eastern and western *S. o’connori* is further supported by results of the phylogenetic analyses (both the NJ and ML trees) of the mtDNA sequences. Fishes from the western region first grouped with the outgroups (*S. waltoni* and *S. macropogon*), and then grouped with fishes of the eastern region. This suggests that the fish in the eastern region are ancestral to, and different from, the fish in the western region.

Consistent with the molecular interpretation presented above, pronounced morphological differences exist between western and eastern individuals of *S. o’connori*^16^. The pharyngeal bone of eastern *S. o’connori* is narrower than that of the western fish, and barbel length of eastern individuals is longer than that of western fishes^16^. In addition, there are three or four rows of pharyngeal teeth observed in eastern *S. o’connori*, but only four rows of pharyngeal teeth in western fishes^16^. The type specimen of *S. o’connori* was collected at Shigatse in the western region of the YLTR^35^, so based on precedence, fishes from this area should be viewed as *S. o’connori*. Fish from the eastern region (PLTR) that are nominally *S. o’connori* require detailed study to validate their systematic status and their taxonomy.

The possibility exists, as noted above, of unidirectional gene flow from the west to the east. Thus, fish at Bomi may, in principle, be able to interbreed with fish from the western populations if such fish are able to survive the journey down the many waterfalls from the YLTR to the PLTR. The mtDNA sequencing data is not well suited to test the suggestion of hybridisation because of the maternal mode of inheritance (i.e., if hybrids are formed then they appear to be only between male western fishes and female eastern fishes). The nDNA codominant microsatellites are more informative as markers of hybridization. The majority of the Bomi fish exhibit multilocus genotypes that are quite distinct from those of the western fishes, suggesting that hybridization is not occurring. However, two Bomi fish exhibit multilocus genotypes that are intermediate between eastern and western population-specific multilocus genotypes, and one fish exhibits a multilocus genotype that is similar to genotypes of western fishes (e.g., Fig. 8). At this stage it is not possible to completely rule out hybridization, although in our view it is unlikely to occur for reasons of distance and barriers to gene flow from the west to the east. Further examination of the Bomi population, as well as other eastern populations is required to better understand if hybridization is occurring, at what sort of rate, and what the consequences are for fishes in the west and east of the QTP.

Revision of the specific status on the *S. o’connori* in the western and eastern regions would change the present situation whereby *S. o’connori* is the only species of endemic *Schizothorax* fish that is distributed in both the western and eastern drainages of the YLTR^16^ so that no species occurs in both the eastern and western regions. In addition, revision of the specific status of *S. o’connori* in the western and eastern regions is likely to result in the recognition of a new subspecies or species in the east that requires specific management and conservation. Clarification of the taxonomic status of the east *S. o’connori* population requires that such work encompasses molecular approaches (e.g. mtDNA COI and nDNA ITS analysis and perhaps SNPs development) as well as morphological analysis, physiological and environmental assessments, and the integration of all findings to help determine the likely status of eastern populations of *S. o’connori*. Ongoing threats to the river systems of the QTP such as pollution, over-fishing and the introduction of non-native species make this work a priority if we are to better understand the phylogeography of the fishes of this biodiversity hotspot.

## Conclusion

Combined analyses of mtDNA and nDNA variation provide evidence that population genetics and dynamics of *S. o’connori* are profoundly influenced by the uplifts of QTP and Quaternary climatic oscillations. The relevance of these findings for *S. o’connori* to other fishes on the QTP, in particular schizothoracine fishes, remains to be determined, as does the taxonomic status of *S. o’connori* from the eastern (PLTR) system which is substantially different from putative conspecifics in the west. In terms of conservation and fisheries management, regardless of taxonomic status, the fishes in the east and west are very clearly different evolutionary units, and must be managed and protected as such.

## Methods

### Ethics statement

No specific permits were required for the field studies described here. We confirm that the study locations were not privately owned or protected, and the field sampling activities did not involve endangered or protected species beyond the focal species. The animal research oversight committee of Huazhong Agricultural University (HZAU) had knowledge of the fish sampling plans prior to their approval of the present animal research protocol. All experimental protocols were approved by the Institutional Animal Care and Use Committee of HZAU, the methods were carried out in accordance with the approved guidelines.

### Sampling and DNA extraction

During the 2012 to 2014, fin clips were obtained from fish at six locations in the middle reaches of the YLTR in the western drainages (above the YTGC) and one location in the tributary of the PLTR in the east drainage (below the YTGC), Tibet (Fig. 1; Supplementary Table S5). Fin clips were collected and preserved in 95% ethanol at −20 °C until DNA extraction. Genomic DNA was extracted using the phenol-chloroform protocol.

### Mitochondrial and microsatellite DNA amplification

DNA from 168 individuals (Table 5) was used to amplify complete mtDNA Cyt *b* (1141 bp) sequences and partial mtDNA CR (~714 bp) sequences following Guo *et al*.^19^. For Cyt *b*, we used primers L14724 (5′-GACTTGAAAAACCACCGTTG-3′) and H15915 (5′-CTCCGATCTCCGGATTACAAGAC-3′), which were used in He & Chen^13^. For CR, we designed a primer pair DL-F (5′-ACTCTCACCACTGGCTCC-3′) and DL-R (5′- GACTCATCTTAGCATCTTCAG-3′) for amplification based on the mitogenome sequence of *S. o’connori* (GenBank accession no. KC513575). PCR products were sequenced on an ABI 3730xl.

For microsatellite analysis, a total of 322 individuals (Supplementary Table S5) were genotyped at 12 microsatellite markers^36^,^37^,^38^,^39^ in four multiplex reactions. These markers were selected based on length, annealing temperature, and scoring performance and primers were synthesised with fluorescent dyes (FAM, HEX or TAMRA, Applied Biosystems) at the 5′ ends (Supplementary Table S4). PCR amplification followed Guo *et al*.^38^. Capillary separation and allele size scoring of microsatellite loci were performed as described in Wei *et al*.^40^.

### Analysis of mitochondrial DNA (mtDNA) sequence data

Sequences of mtDNA were multiply aligned using Clustal X 2.0^41^ after manual editing and checking using DNASTAR software^42^. A combined sequence was constructed by concatenation of Cyt *b* + CR to combine the phylogenetic signal to maximise the possibility of finding an optimal topology.

Population genetic analysis was carried out on Cyt *b*, CR and Cyt *b* + CR sequences, respectively. Haplotype diversity (*Hd*), nucleotide diversity (*π*), number of segregating sites (*S*), number of haplotypes (*h*) and number of nucleotide differences (*K*) were estimated for the whole data set and for each population using Arlequin 3.11^43^ and DNASP 5.0^44^. Popart^45^ was used to reconstruct phylogenetic relationships among haplotypes with a median-joining network method. Because Cyt *b* + CR provided most information we focus primarily on the concatenated sequence.

Hierarchical analysis of molecular variance (AMOVA) was performed to test for genetic differentiation between regions (west/east) or among populations using Arlequin 3.11. Statistical significance of fixation indices (*Φ*) was tested with 10,000 permutations. Genetic distances within/between populations based on the K2P model were calculated using MEGA 5.0^46^. GenAlEx 6.5^47^ was used to examine isolation by distance (IBD) by testing for a linear relationship between pairwise population *Φ*_ST_/(1 − *Φ*_ST_) statistics and log (geographical distances (km)) for all seven populations, and for the six western populations (Supplementary Table S6).

To determine relationships among populations and if there was any evidence of historical dispersal, phylogenetic analysis was carried out on Cyt *b*, CR and the concatenated data set (Cyt *b* + CR) independently using neighbour-joining (NJ) and maximum likelihood (ML) in MEGA 5.0. The best-fitting nucleotide substitution model with the lowest Bayesian information criterion (BIC) score was determined using MEGA 5.0. ML analyses were performed under the TN93 + G (Cyt *b*), T92 + G + I (CR) and HKY + G + I (Cyt *b* + CR) model. In the NJ analysis, the K2P model was used. The reliability of nodes was assessed using 1000 bootstrap replicates. The congeneric species *S. waltoni* (GenBank accession no. JX202592), *S. macropogon* (GenBank accession no. KC020113) and *Schizopygopsis younghusbandi* (GenBank accession no. KC351895) were designated as outgroups.

A continuous phylogenetic analysis was conducted using BEAST 2.3.2^48^ to incorporate spatial and temporal components into the phylogeny. Analyses were performed with Cyt *b* and the concatenated data set (Cyt *b* + CR) independently. The clock model and tree prior model were compared by calculating Bayes factor (BF). BF was estimated according to the marginal likelihood estimated by using the smoothed harmonic mean estimator^49^, as implemented in the Tracer 1.5 (http://beast.bio.ed.ac.uk/Tracer). In this study, we tested two clock models: a strict clock and an uncorrelated lognormal relaxed clock. Two tree prior models, Yule process speciation and coalescent Bayesian Skyline, were also compared using BF calculation. As a result, all the data sets were analysed using a HKY model of nucleotide substitution under an uncorrelated exponential relaxed clock with a coalescent Bayesian Skyline tree prior model. The data of each model are shown in Supplementary Tables S7 and S8. Unfortunately, the timing of the diversification processes cannot be inferred with accuracy due to a lack of fossils in *S. o’connori* and hindered by the exhibition of different mutation rates by different lineages, therefore the molecular dating based on a fixed rate of mutation is not particularly suitable in this fish but at least gives us an idea about the potential age of diversification events. Nucleotide substitution rates for Cyt *b* and CR were assumed to be 1.0% and 3.6% per site per million years according to the previous study^50^,^51^. We assumed the previously established average nucleotide substitution rate of 1.69% per site per million years that has been calibrated for concatenated sequence (Cyt *b* + CR) in schizothoracine fishes^4^,^5^. The Markov chain Monte Carlo (MCMC) analyses were run for 100 million generations, with parameters logged every 20,000 generations. Convergence was assessed from the effective sample size (ESS) after a 15% burn-in using Tracer 1.5. ESS values of parameters of interest above 200 were accepted. Uncertainty in the estimates was indicated by the 95% highest posterior density (95% HPD) intervals. The first 10% of the generations were discarded as burn-in, and the rest were retained as valid samples for further analyses. The maximum clade credibility tree was generated by TreeAnnotator 2.3.2^48^ and was viewed in FigTree 1.4 (http://tree.bio.ed.ac.uk/software/figtree/) with *S. waltoni* and *S. macropogon* used as outgroups.

Historical population dynamics were estimated using coalescent-based Bayesian skyline plots (BSPs)^52^ and mismatch distributions^31^. BSPs were performed using BEAST 2.3.2 to describe demographic history by assessing the time variation of effective population size. All the sequences data and similar settings as above were used in the BSPs analysis. The results of BSPs were visualized in Tracer 1.5, which summarized the posterior distribution of population size over time. Mismatch distributions were implemented in Arlequin 3.11. In addition, Fu’s *F*s^53^ tests were used to determine if the two mtDNA sequences conformed to the expectations of neutrality. The sum of squared deviations (*SSD*) and the raggedness index (*r*)^54^ were estimated in Arlequin 3.11 to determine if sequences deviated significantly from a model of population expansion. If evidence of population expansion was found, the possible number of generations since population expansion (t) was estimated from the equation t = τ/2u, where u is neutral mutation rate for the entire sequence per generation and is calculated as u = 2 μkT_gen_, where μ is the mutation rate per nucleotide per year or million years; k is the average sequence length of the mtDNA region under study; and T_gen_ is the generation time in years^55^. The approximate time of population expansion in years (T) was calculated by multiplying the number of generations (t) by the generation time (T_gen_). Generation time for *S. o’connori* was estimated to be 10 years based on approximate time at which females become mature^56^. We only consider the female age because the mitochondria of males are not transmitted to offspring^57^.

### Analysis of microsatellite DNA data

Because it is unknown if *S. o’connori* is an autotetraploid or an allotetraploid species the microsatellite data were treated as phenotypic data (describing the identity and presence of observed bands, but not band copy numbers) and were analysed in two ways. First, all bands at each locus were combined to form multilocus allelic phenotypes for each individual. These phenotypes record the presence and identity of each allele, but are not genotypes because they contain no information about allele copy numbers unless individuals are homozygous (four identical alleles giving one band) or have four different alleles. Second, each microsatellite locus was treated as dominant and then the multilocus allelic phenotypes were transformed into binary - presence (1) or absence (0) - for each individual. Although there is some information loss using binary data, such data are free from unrealistic assumptions about allelic identities and may be used in analysis of molecular variance (AMOVA). These approaches are routinely employed to evaluate genetic diversity and population structure of polyploidy species^58^.

Binary allelic diversity was measured as total number of inferred bands (Total bands) and number of bands unique to a single population (Private bands) and they were calculated using GenAlEx 6.5. Nei’s gene diversity index (*H*), Shannon’s information index (*I*), and the percentage of polymorphic loci (*PPL*) were calculated using POPGENE^59^ and GenAlEx 6.5. Estimation of genetic differentiation (*F*_ST_) and analysis of molecular variance (AMOVA) were performed using Arlequin 3.11.

Estimation of genetic distance between populations of polyploid organisms is problematical because there is no accepted methodology for the calculation of genetic distance with binary data^58^. It is however, reasonable to use genetic distance indices as descriptive measures, because banding patterns in polyploids specify phenotypes^58^. Thus, Nei’s unbiased genetic distance was calculated based on binary matrix data using GenAlEx 6.5. The relationships between individuals and populations (PCoA) based on pairwise Euclidean distances were also performed using GenAlEx 6.5. Isolation by distance (IBD) was assessed as previously described. Using the original genotypic data, a Bayesian clustering method implemented in STRUCTURE 2.3.4^60^ was used to determine the most likely number of genetic clusters, regardless of site of collection. Given the nature of the polyploidy microsatellite data set it cannot be known if assumptions for STRUCTURE such as Hardy-Weinberg equilibrium and no linkage disequilibrium are met, so we employ this approach as an exploratory analysis. The admixture model was chosen, allele frequencies were assumed to be correlated and analyses were conducted with a burn-in of 10,000 and followed by 100,000 MCMC repetitions. Ten independent runs were carried out for each cluster set (*K*) from 1 to 7. Further, we used the Δ*K* metric^60^ to determine the statistically most supported number of clusters as implemented in the web interface STRUCTURE HARVESTER (http://taylor0.biology.ucla.edu/structureHarvester/).

## Additional Information

**How to cite this article**: Guo, X.-Z. *et al*. Phylogeography and population genetics of *Schizothorax o’connori*: strong subdivision in the Yarlung Tsangpo River inferred from mtDNA and microsatellite markers. *Sci. Rep*. **6**, 29821; doi: 10.1038/srep29821 (2016).

## Supplementary Material

Supplementary Information

## Figures and Tables

**Table 1:** Hierarchical analysis of molecular variance (AMOVA) of *Schizothorax o’connori* population genetic variation based on Cyt *b*, CR and Cyt*b* + CR data set, as well as microsatellite variation.

Source of variation	df	Variance component	Percentage of variation	Fixation index
**Cyt** ***b***				
Among populations	6	3.279	63.89	*Φ*_ST_ = 0.639***
Within populations	161	1.853	36.11	
Between regions (west/east)	1	11.385	85.84	*Φ*_CT_ = 0.858
Among populations within regions	5	0.026	0.19	*Φ*_SC_ = 0.014
Within populations	161	1.853	13.97	*Φ*_ST_ = 0.860***
**CR**				
Among populations	6	2.406	51.42	*Φ*_ST_ = 0.514***
Within populations	161	2.273	48.58	
Between regions (west/east)	1	8.130	77.53	*Φ*_CT_ = 0.775
Among populations within regions	5	0.083	0.79	*Φ*_SC_ = 0.035**
Within populations	161	2.273	21.68	*Φ*_ST_ = 0.783***
**Cyt** ***b*** + **CR**				
Among populations	6	5.685	57.94	*Φ*_ST_ = 0.579***
Within populations	161	4.126	42.06	
Between regions (west/east)	1	19.515	82.17	*Φ*_CT_ = 0.822
Among populations within regions	5	0.109	0.46	*Φ*_SC_ = 0.026*
Within populations	161	4.126	17.37	*Φ*_ST_ = 0.826***
**Microsatellites**				
Among populations	6	1.694	8.37	*F*_ST_ = 0.084***
Within populations	315	18.545	91.63	
Between regions (west/east)	1	2.016	9.30	*F*_CT_ = 0.093
Among populations within regions	5	1.118	5.16	*F*_SC_ = 0.057***
Within populations	315	18.545	85.54	*F*_ST_ = 0.145***

^*^P < 0.05, **P < 0.01, ***P < 0.001.

**Table 2:** Population expansion time for the six populations in the western region based on mtDNA data set.

Population	Cyt *b*		CR		Cyt *b* + CR	
	τ	T	τ	T	τ	T
Shigatse	6.178	0.135	6.816	0.066	14.248	0.114
Zhaxue	7.117	0.156	6.676	0.065	12.152	0.097
Quxu	5.395	0.118	7.23	0.070	11.928	0.095
Shannan	5.816	0.127	8.141	0.079	13.09	0.104
Mainling	6.219	0.136	6.338	0.062	10.941	0.087
Paizhen	5.938	0.130	6.863	0.067	11.711	0.093
Mean	6.111	0.134	7.011	0.068	12.345	0.098

τ, the mode of mismatch distribution; T, approximate time of expansion in million years before present (Ma).

**Table 3:** Nuclear diversity statistics for seven populations of *Schizothorax o’connori* based on analysis of 12 microsatellite loci.

Population	Total bands	Private bands	*PPL*	*H*	*I*
Shigatse	220	5	61.28%	0.071	0.128
Zhaxue	231	7	64.07%	0.073	0.132
Quxu	213	4	59.33%	0.069	0.124
Shannan	211	13	58.77%	0.071	0.128
Mainling	228	13	63.51%	0.071	0.128
Paizhen	221	11	61.28%	0.069	0.125
Bomi	145	18	40.11%	0.062	0.107
Mean	210	10.14	58.33%	0.069	0.125
Total	359	71.00	—	—	—

*PPL*, percentage of polymorphic loci; *H*, Nei’s gene diversity index; *I*, Shannon’s information index.

**Table 4:** Matrix of pairwise *F*_ST_ values (below diagonal) and Nei’s unbiased genetic distances (above diagonal) among seven *Schizothorax o’connori* populations based on variation at 12 microsatellite loci.

Population	Shigatse	Zhaxue	Quxu	Shannan	Mainling	Paizhen	Bomi
Shigatse	—	0.002	0.006	0.007	0.005	0.000	0.009
Zhaxue	**0.035**	—	0.002	0.002	0.002	0.002	0.006
Quxu	**0.101**	**0.045**	—	0.001	0.001	0.006	0.008
Shannan	**0.104**	**0.038**	0.007	—	0.001	0.007	0.009
Mainling	**0.085**	**0.032**	**0.014**	**0.015**	—	0.005	0.009
Paizhen	0.003	**0.034**	**0.102**	**0.106**	**0.082**	—	0.008
Bomi	**0.165**	**0.131**	**0.164**	**0.161**	**0.152**	**0.144**	—

Significant *F*_ST_ values after Bonferroni correction for multiple testing are in bold (*α* = 0.05).
