# Supplementary material for: Phylogeography and population genetics of Schizothorax o’connori: strong subdivision in the Yarlung Tsangpo River inferred from mtDNA and microsatellite markers
Source: Sci Rep. 2016 Jul 18;6:29821. doi: 10.1038/srep29821 (PMC4947931; doi:10.1038/srep29821)
Supplement: Supplementary Information [file srep29821-s1.pdf]

Phylogeography and population genetics of *Schizothorax o'connori*: strong subdivision in the Yarlung Tsangpo River inferred from mtDNA and microsatellite markers

Xiang-Zhao Guo[1, 2], Gui-Rong Zhang[1, 2], Kai-Jian Wei[1, 2, *], Ruo-Jin Yan[1, 2], Wei Ji[1, 2], Rui-Bin Yang[1, 2], Qi-Wei Wei[3] & Jonathan P. A. Gardner[1, 2, 4]

[1] - Key Laboratory of Freshwater Animal Breeding, Ministry of Agriculture, College of Fisheries, Huazhong Agricultural University, Wuhan 430070, P. R. China

[2] - Freshwater Aquaculture Collaborative Innovation Center of Hubei Province, Wuhan 430070, P. R. China

[3] - Key Laboratory of Freshwater Biodiversity Conservation, Ministry of Agriculture, Yangtze River Fisheries Research Institute, Chinese Academy of Fishery Sciences, Wuhan 430223, P. R. China

[4] - School of Biological Sciences, Victoria University of Wellington, P O Box 600, Wellington 6140, New Zealand

* - **Correspondence**:

Kai-Jian Wei, College of Fisheries, Huazhong Agricultural University, Wuhan 430070, P. R. China. Tel: 86 27 87282113, Fax: 86 27 87282114. E-mail: kjwei@mail.hzau.edu.cn

Figures

Figure S1. NJ (a) and ML (b) phylogenetic trees of *Schizothorax o'connori*, based on Cyt *b* haplotypes. The numbers above the branches correspond to bootstrap support > 50% obtained in the NJ and ML analyses.

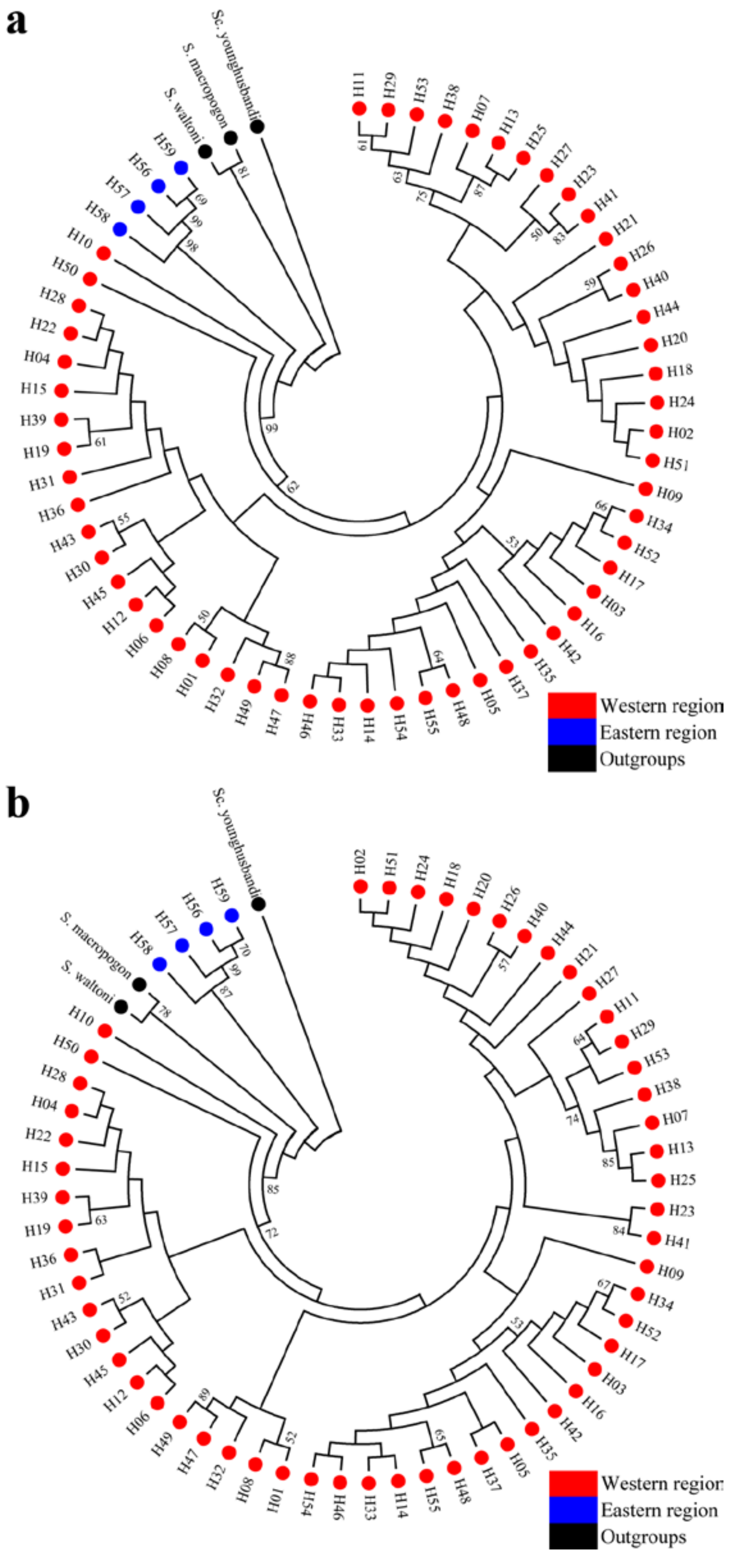

Figure S2. NJ (a) and ML (b) phylogenetic trees of *Schizothorax o'connori*, based on CR haplotypes. The numbers above the branches correspond to bootstrap support > 50% obtained in the NJ and ML analyses.

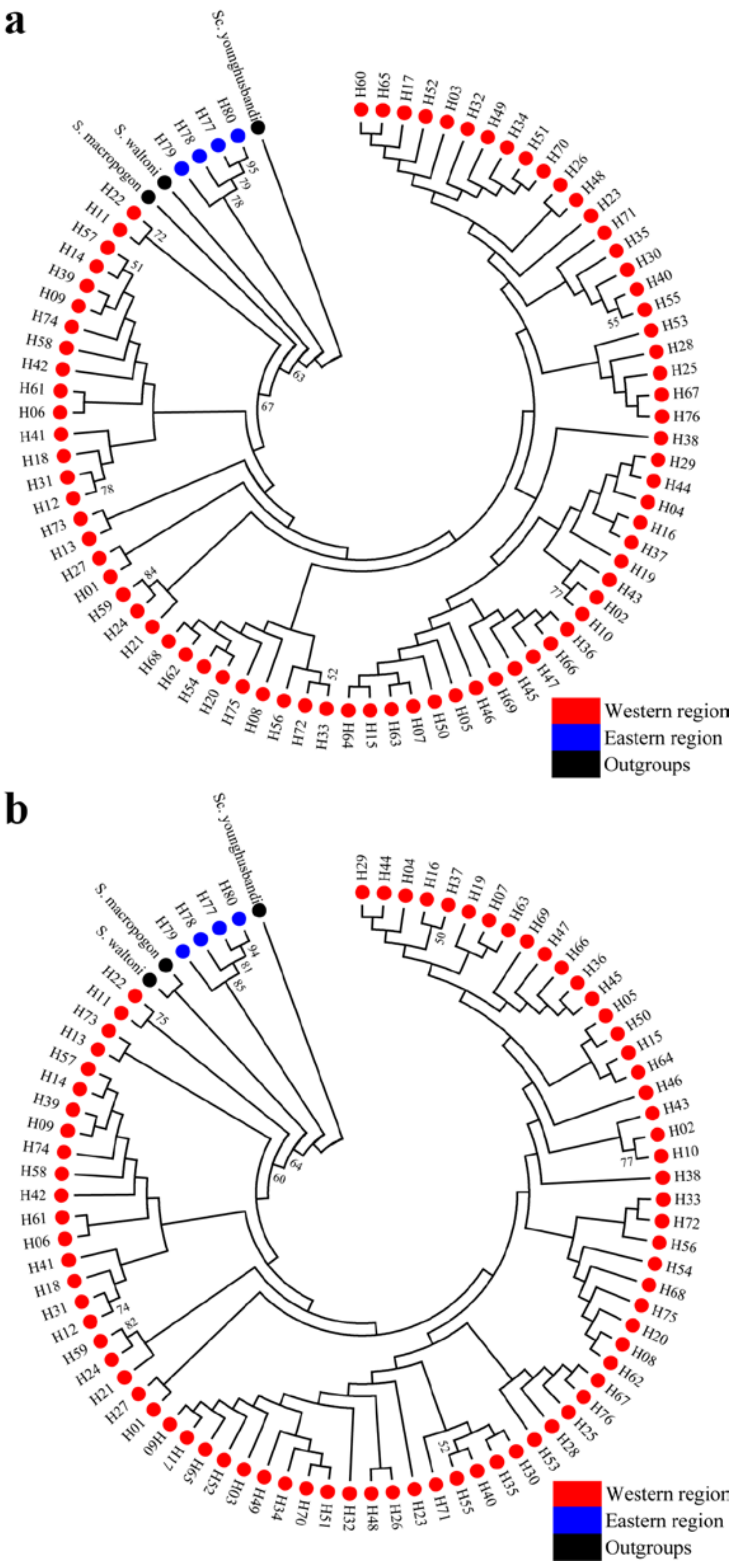

Figure S3. Median-joining network of mtDNA Cyt *b* haplotypes from seven populations of *Schizothorax o'connori*. The circle size of haplotype denotes the number of observed individuals. Colors correspond to different regions. White circles represent intermediate haplotypes not observed.

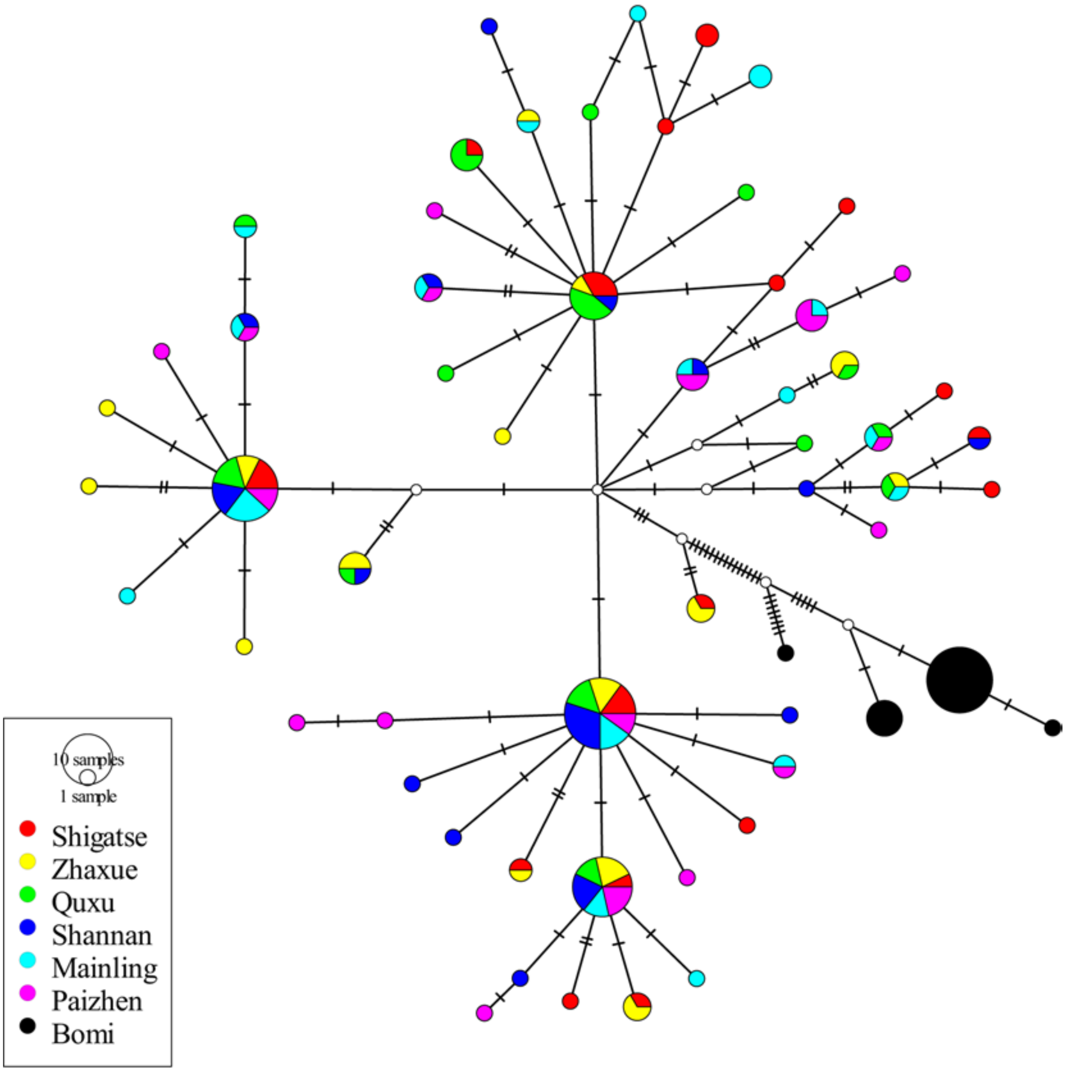

Figure S4. Median-joining network of mtDNA CR haplotypes from seven populations of *Schizothorax o'connori*. The circle size of haplotype denotes the number of observed individuals. Colors correspond to different regions. White circles represent intermediate haplotypes not observed.

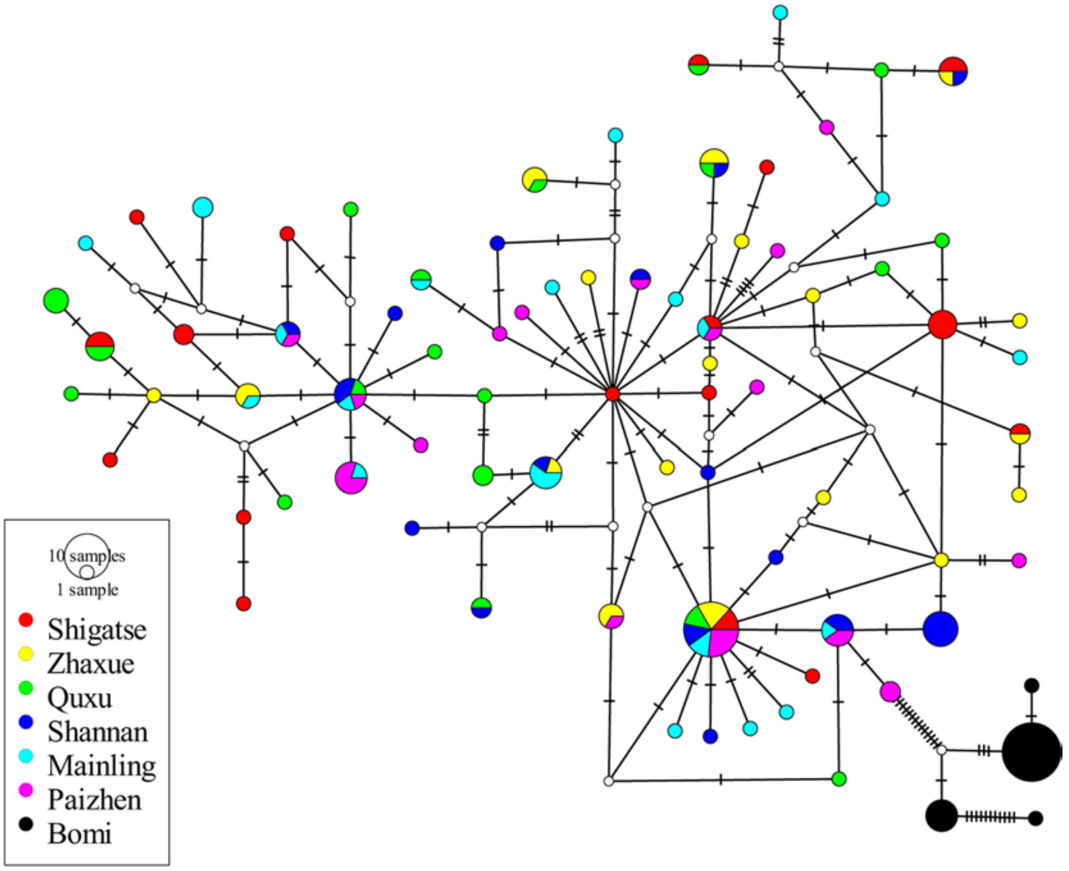

Figure S5. Bayesian phylogenetic tree for *Schizothorax o'connori*, based on Cyt *b* haplotypes. The numbers above the branches are the estimates of divergence times (million years, Ma) within *Schizothorax o'connori* for the major nodes by BEAST analysis. Blue shaded bars indicate the 95% highest posterior density (HPD) for node ages and scale bars represents time in millions of years from the present day. Western and eastern clades are denoted by red and blue lines, respectively.

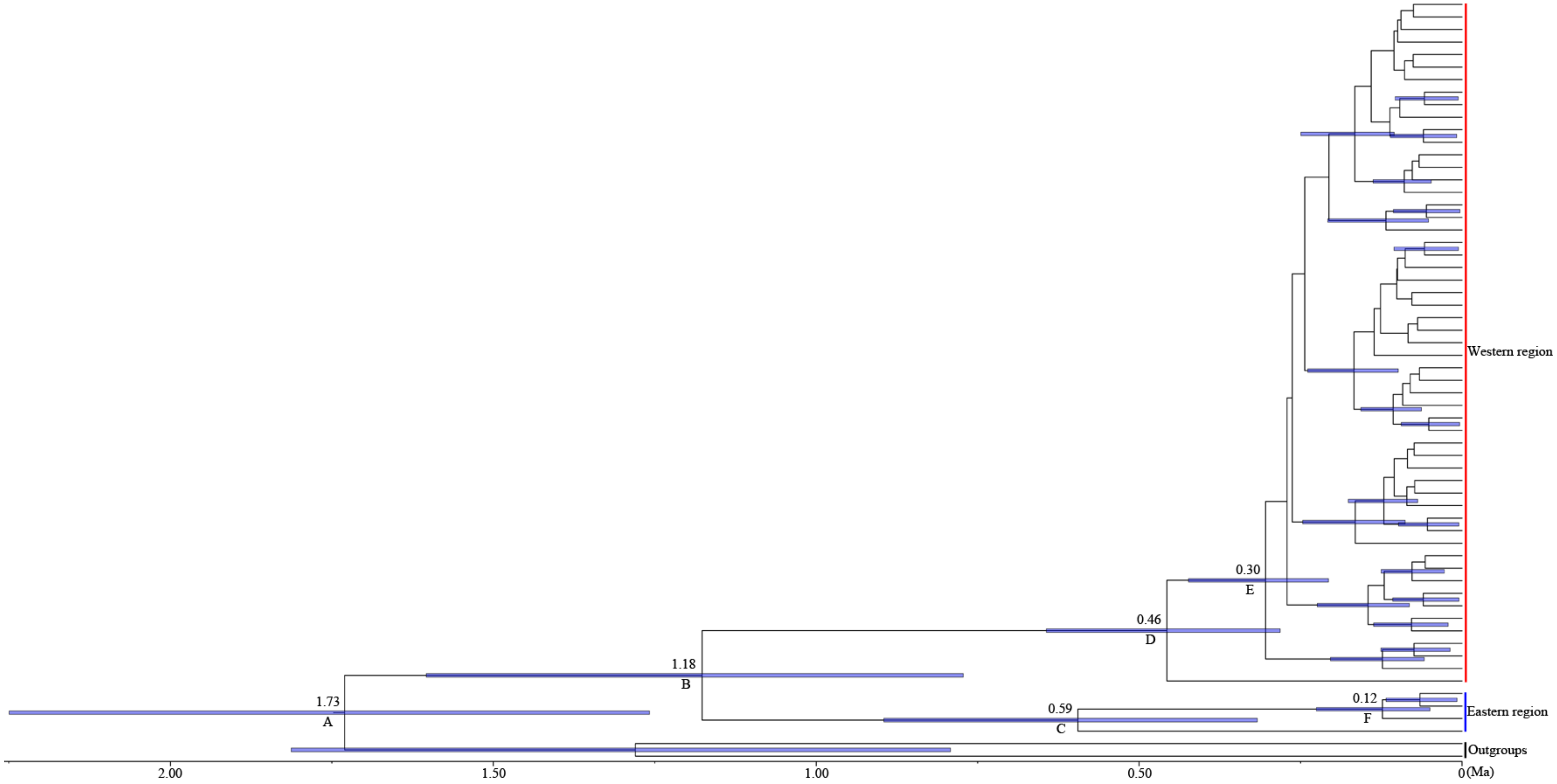

Table S1. Haplotype distributions in seven populations of *Schizothorax o'connori* based on Cyt *b*, CR and Cyt *b* + CR data set

Sequence	Population	Haplotype (individual numbers)
Cyt *b*	Shigatse	H01(1), **H02(3)**, H03(1), H04(3), **H05(3)**, H06(2), H07(1), H08(1), H09(1), H10(1), H11(1), H12(1), H13(1), H14(1), H15(1), H16(1), **H17(1)**
	Zhaxue	**H02(2)**, H04(1), **H05(3)**, H09(1), H10(2), H16(2), **H17(3)**, H18(1), H19(1), H20(1), H21(2), H22(1), H23(2), H24(1), H25(1)
	Quxu	**H02(3)**, H04(4), **H05(3)**, H15(3), **H17(2)**, H21(1), H23(1), H25(1), H26(1), H27(1), H28(1), H29(1), H30(1), H31(1)
	Shannan	**H02(3)**, H04(1), **H05(6)**, H13(1), **H17(3)**, H21(1), H32(1), H33(1), H34(1), H35(1), H36(1), H37(1), H38(1), H39(1), H40(1)
	Mainling	**H02(4)**, **H05(3)**, **H17(2)**, H19(1), H25(1), H26(1), H29(1), H32(1), H36(1), H40(1), H41(1), H42(1), H43(1), H44(1), H45(2), H46(1), H47(1)
	Paizhen	**H02(2)**, **H05(2)**, **H17(3)**, H29(1), H32(2), H36(1), H40(1), H46(1), H47(3), H48(1), H49(1), H50(1), H51(1), H52(1), H53(1), H54(1), H55(1)
	Bomi	H56(17), H57(5), H58(1), H59(1)

	Shigatse	H01(1), H02(1), H03(1), H04(1), H05(1), H06(4), H07(2), H08(1), H09(2), H10(1), H11(1), H12(1), H13(1), H14(1), H15(1), H16(2), **H17(2**)
	Zhaxue	H09(1), H12(1), **H17(3)**, H18(1), H19(2), H20(1), H21(2), H22(1), H23(2), H24(2), H25(1), H26(1), H27(1), H28(1), H29(1), H30(1), H31(1), H32(1)
	Quxu	H14(1), H16(2), **H17(2)**, H21(1), H24(1), H33(1), H34(1), H35(2), H36(1), H37(3), H38(1), H39(1), H40(1), H41(1), H42(1), H43(1), H44(1), H45(1), H46(1)
CR	Shannan	H09(1), **H17(2)**, H21(1), H30(1), H40(1), H45(2), H47(1), H48(1), H49(6), H50(1), H51(2), H52(1), H53(1), H54(1), H55(1), H56(1)
	Mainling	H13(1), **H17(2)**, H19(1), H30(3), H33(1), H45(1), H50(1), H51(1), H57(1), H58(1), H59(1), H60(1), H61(1), H62(1), H63(1), H64(2), H65(1), H66(1), H67(1), H68(1)
	Paizhen	H13(1), **H17(4)**, H23(1), H45(1), H50(1), H51(2), H54(1), H66(4), H69(1), H70(2), H71(1), H72(1), H73(1), H74(1), H75(1), H76(1)
	Bomi	H77(15), H78(5), H79(1), H80(1)

	Shigatse	H17(1), **H23(1)**, H50(1), H57(1), H65(1), H66(1), H67(1), H68(1), H69(1), H70(3), H71(2), H72(1), H73(1), H74(1), H75(1), H76(1), H77(1), H78(1), H79(1), H80(1), H81(1)

	Zhaxue	H13(1), **H23(2)**, H28(1), H59(2), H61(2), H76(1), H94(1), H95(1), H96(1), H97(1), H98(2), H99(1), H100(1), H101(1), H102(1), H103(1), H104(1), H105(1), H106(1), H107(1)
	Quxu	H15(1), **H23(2)**, H46(1), H47(2), H48(1), H49(2), H50(1), H51(1), H52(1), H53(1), H54(1), H55(1), H56(1), H57(1), H58(1), H59(1), H60(1), H61(1), H62(1), H63(1), H64(1)
Cyt *b* + CR	Shannan	H07(1), H13(1), **H23(1)**, H53(1), H59(1), H63(1), H79(1), H82(1), H83(1), H84(1), H85(4), H86(1), H87(1), H88(1), H89(1), H90(1), H91(1), H92(2), H93(1)
	Mainling	H06(1), H07(1), H08(1), H(09), H10(1), H11(1), H12(1), H13(1), H14(1), H15(1), H16(1), H17(1), H18(1), H19(1), H20(1), H21(1), H22(1), **H23(1)**, H24(1), H25(1), H26(1), H27(1), H28(1), H29(1)
	Paizhen	H07(1), H12(1), **H23(3)**, H30(1), H31(1), H32(2), H33(2), H34(1), H35(1), H36(1), H37(1), H38(1), H39(1), H40(1), H41(1), H42(1), H43(1), H44(1), H45(1)
	Bomi	H01(16), H02(5), H03(1), H04(1), H05(1)

Bold indicates haplotypes that are shared by the six populations in the western region.

Table S2. Genetic diversity indices and results of mismatch distribution and neutrality tests based on Cyt *b*, CR and Cyt *b* + CR data set

Sequence	Population	n	*h*	*S*	*Hd*	*π*	*K*	*SSD*	*r*	Distribution	*Fs*
Cyt *b*	Shigatse	24	17	28	0.964	0.00382	4.355	0.003	0.015	unimodal	**-8.809**
	Zhaxue	24	15	28	0.960	0.00413	4.714	0.003	0.015	unimodal	**-5.204**
	Quxu	24	14	21	0.942	0.00306	3.496	0.009	0.034	unimodal	**-5.821**
	Shannan	24	15	22	0.924	0.00284	3.239	0.007	0.027	unimodal	**-7.879**
	Mainling	24	17	23	0.960	0.00360	4.112	0.018	0.046	unimodal	**-9.308**
	Paizhen	24	17	24	0.967	0.00361	4.123	0.019	0.044	unimodal	**-9.285**
	Bomi	24	4	16	0.471	0.00167	1.902	**0.327**	0.376	multimodal	1.924
	Total	168	59	84	0.954	0.00820	9.360	0.055	0.079	multimodal	-6.340
CR	Shigatse	24	17	22	0.964	0.00708	5.051	0.003	0.018	unimodal	**-7.584**
	Zhaxue	24	18	29	0.975	0.00700	4.841	0.016	0.039	unimodal	**-9.376**
	Quxu	24	19	26	0.978	0.00765	5.378	0.004	0.019	unimodal	**-10.456**
	Shannan	24	16	22	0.935	0.00621	4.348	0.019	0.032	unimodal	**-7.039**
	Mainling	24	20	28	0.982	0.00706	5.033	0.007	0.027	unimodal	**-13.422**
	Paizhen	24	16	23	0.949	0.00601	4.286	0.002	0.014	unimodal	**-7.292**

	Bomi	24	4	16	0.471	0.00360	2.565	**0.325**	0.409	multimodal	2.975
	Total	168	80	67	0.975	0.01213	8.649	0.054	0.080	multimodal	-7.456
Cyt *b* + CR	Shigatse	24	21	50	0.986	0.00507	9.406	0.005	0.015	unimodal	**-9.414**
	Zhaxue	24	20	57	0.986	0.00524	9.554	0.012	0.026	unimodal	**-7.301**
	Quxu	24	21	47	0.989	0.00483	8.873	0.003	0.008	unimodal	**-9.852**
	Shannan	24	20	44	0.975	0.00414	7.587	0.014	0.018	unimodal	**-9.262**
	Mainling	24	24	51	1.000	0.00493	9.145	0.004	0.010	unimodal	**-18.029**
	Paizhen	24	20	47	0.978	0.00454	8.409	0.007	0.019	unimodal	**-8.466**
	Bomi	24	5	32	0.529	0.00241	4.467	**0.382**	0.295	multimodal	4.054
	Total	168	107	151	0.984	0.00971	18.009	0.061	0.056	multimodal	-8.324

n, number of samples, *h*, number of haplotypes; *S*, number of segregating sites; *Hd*, haplotype diversity; π, nucleotide diversity; *K*, number of nucleotide differences; *SSD*, sum of the squared differences under expansion model; *r*, raggedness index; Distribution, the shape of mismatch distribution; *Fs*, Fu's *Fs* test statistic; numbers in bold indicate statistically significant results ($P < 0.05$).

Table S3. Pairwise Φ_{ST} (below diagonal), average Kimura 2-parameter (K2P) genetic distance values within populations (shown in bold along diagonal) and between pairs of populations (above diagonal) based on Cyt *b*, CR and Cyt *b* + CR data set

Sequence	Population	Shigatse	Zhaxue	Quxu	Shannan	Mainling	Paizhen	Bomi
Cyt *b*	Shigatse	**0.0039**	0.0041	0.0034	0.0034	0.0037	0.0039	0.0235
	Zhaxue	0.0092	**0.0042**	0.0037	0.0035	0.0039	0.0040	0.0238
	Quxu	-0.0131	0.0164	**0.0031**	0.0031	0.0034	0.0036	0.0233
	Shannan	0.0059	-0.0046	0.0340	**0.0029**	0.0032	0.0033	0.0236
	Mainling	-0.0052	-0.0023	-0.0015	-0.0025	**0.0036**	0.0037	0.0236
	Paizhen	0.0249	0.0226	0.0525*	0.0017	0.0051	**0.0036**	0.0239
	Bomi	0.8784*	0.8727*	0.8940*	0.9003*	0.8835*	0.8845*	**0.0017**
CR	Shigatse	**0.0072**	0.0073	0.0074	0.0071	0.0073	0.0070	0.0303
	Zhaxue	0.0319	**0.0069**	0.0077	0.0068	0.0072	0.0068	0.0299
	Quxu	-0.0017	0.0479	**0.0077**	0.0074	0.0076	0.0073	0.0308
	Shannan	0.0571	0.0361	0.0660	**0.0062**	0.0068	0.0062	0.0287
	Mainling	0.0176	0.0172	0.0201	0.0096	**0.0072**	0.0065	0.0299
	Paizhen	0.0512*	0.0469*	0.0568	0.0029	-0.0143	**0.0061**	0.0289

	Bomi	0.8143*	0.8140*	0.8080*	0.8207*	0.8122*	0.8252*	**0.0037**
	Shigatse	**0.0051**	0.0053	0.0050	0.0048	0.0051	0.0051	0.0261
	Zhaxue	0.0213	**0.0052**	0.0052	0.0048	0.0051	0.0051	0.0261
	Quxu	-0.0065	0.0343	**0.0048**	0.0047	0.0050	0.0050	0.0262
Cyt *b* + CR	Shannan	0.0350	0.0179	0.0533	**0.0041**	0.0046	0.0044	0.0255
	Mainling	0.0073	0.0082	0.0112	0.0044	**0.0050**	0.0048	0.0260
	Paizhen	0.0388	0.0352	0.0549	0.0024	-0.0051	**0.0046**	0.0258
	Bomi	0.8500*	0.8469*	0.8552*	0.8660*	0.8522*	0.8591*	**0.0025**

* $P < 0.05$ after Bonferroni correction.

Table S4. Details of the 12 microsatellite primers used for genotyping

Locus name	GenBank accession no.	Primer sequence (5'-3') (F, forward; R, reverse)	Repeat motif	T_a (°C)	Multiplex	Fluorescent labelling	N_A	N_P	Band size range (bp)
JLL 01	KC880056	F: TCATTTACACAGTAGGGAGC<br>R: CAGTTAGAGGTGACGGAAG	$(AC)_4$…$(TCCTC)_4$	54	3	TAMRA	24	1 – 4	208 – 287
JLL 21	KC880076	F: GACAGACAGAAAGACCAGAGA<br>R: GGTAAACTATCCCAAAATCAT	$(AGAT)_{12}$	56	3	FAM	17	1 – 4	101 – 163
LLK27	KC907359	F: ATCATTCAAAGGTCACTCGT<br>R: TCCACAGAGATGCCAAAG	$(TAGA)_8$	58	4	FAM	15	1 – 4	128 – 165
LLK28	KC907360	F: GAACGAGAAAGTTAAAGGTC<br>R: AGGAGTGGTCAGTGCTTC	$(ATAG)_{21}$	55	3	HEX	21	1 – 4	174 – 244
Scho01	KC247930	F: TAATGATAATGCCGTGTCGTA<br>R: GAAACAGAAAACAGCCCAGAT	$(TG)_{12}$	57	2	HEX	39	1 – 4	240 – 293
Scho23	KC902766	F: CACACAATCAGTAGGTCAGG<br>R: ACTAGCAGTTTATCTTCTCAGC	$(AGAC)_6$…$(TG)_6$	60	1	FAM	7	1 – 4	230 – 250
Scho24	KC902767	F: ATTTTTCCTCTGCCCATTGA<br>R: TTGTGAACCGTTACACCCCT	$(CTAT)_{17}$…$(GTCT)_8$…$(GTCT)_9$	56	2	FAM	41	1 – 4	169 – 276

Scho26	KC902769	F: GCAAAGCACAAAGGATCT	$(TCTG)_4$…$(TCTA)_7$	58	1	HEX	25	1 – 4	107 – 174
		R: CTGAACCATTACACCCCTA							
Scho27	KC902770	F: CGTCTATTGTCTGCTCATCA	$(ATAG)_{14}$	56	1	TAMRA	36	1 – 4	108 – 190
		R: ATCTGCTTACGCCCCAT							
Scho32	KC902775	F: TGAGCAAAACCACTAACACA	$(AGAT)_5$…$(AGAT)_5$	56	2	TAMRA	32	1 – 4	278 – 377
		R: GACGGCACACATTTCTGA							
Scho40	KC902783	F: TAGAGGAGGATGGGTGAGAA	$(TCTA)_9$	54	4	TAMRA	50	1 – 4	205 – 317
		R: CCAACACTGCGAACGATAG							
Scho42	KC902785	F: ATAAGAGGAAAACAATGCC	$(GATA)_{17}$	56	4	HEX	52	2 – 4	134 – 241
		R: AGACCAATGTGTAACAGTAATG							

T_a, annealing temperature of each primer; N_A, number of bands observed at each locus; N_P, number of bands observed in each tetraploid individual.

Table S5. Geographic location and sample sizes of the seven *Schizothorax o'connori* populations in this study

Population	Code	Alt (m)	Lat	Long	n	N
Shigatse	SG	3842	29.32	88.86	24	47
Zhaxue	ZX	3913	30.07	91.76	24	43
Quxu	QX	3595	29.35	90.73	24	49
Shannan	SN	3554	29.26	91.82	24	41
Mainling	ML	2964	29.19	94.00	24	49
Paizhen	PZ	2922	29.47	94.68	24	47
Bomi	BM	2685	29.92	95.64	24	46
Mean	—	—	—	—	24	46
Total	—	—	—	—	168	322

Alt, altitude (m); Lat, latitude (°N); Long, longitude (°E); n, number of individuals for mtDNA analysis; N, number of individuals for microsatellite DNA analyses.

Table S6. Matrix of pairwise population geographic distances (km)

Population	Shigatse	Zhaxue	Quxu	Shannan	Mainling	Paizhen	Bomi
Shigatse	–						
Zhaxue	292.26	–					
Quxu	181.30	127.69	–				
Shannan	287.13	90.25	106.16	–			
Mainling	498.81	237.59	317.67	211.69	–		
Paizhen	564.03	289.62	382.83	278.13	72.90	–	
Bomi	658.67	374.01	478.73	376.57	178.19	105.37	–

Table S7. Marginal likelihood estimated from different clock and prior tree models based on Cyt *b* and Cyt *b* + CR data set

Sequence	Model code	Clock model	Priors tree model	Ln P(data/model)
Cyt *b*	A	Strict clock	Yule	-2461.541
	B	Strict clock	Bayesian skyline	-2456.175
	C	Lognormal	Yule	-2467.471
	**D**	**Lognormal**	**Bayesian skyline**	**-2455.771**
Cyt *b* + CR	E	Strict clock	Yule	-4678.743
	F	Strict clock	Bayesian skyline	-4673.752
	G	Lognormal	Yule	-4675.900
	**H**	**Lognormal**	**Bayesian skyline**	**-4672.862**

Data and models with bold are used in this study.

Table S8. Bayes Factor (Ln (BF)) calculated from the marginal likelihood of different models based on Cyt *b* and Cyt *b* + CR data set

Sequence	Model comparation	Ln (BF)
Cyt *b*	A/B	-5.366
	A/C	5.930
	A/D	-5.770
	B/C	11.296
	B/D	-0.404
	C/D	-11.700
Cyt *b* + CR	E/F	-4.991
	E/G	-2.843
	E/H	-5.881
	F/G	2.148
	F/H	-0.890
	G/H	-3.038
